# The Sweet Side of Immune Evasion: Role of Glycans in the Mechanisms of Cancer Progression

**DOI:** 10.3389/fonc.2016.00054

**Published:** 2016-03-09

**Authors:** Ana Flávia Fernandes Ribas Nardy, Leonardo Freire-de-Lima, Célio Geraldo Freire-de-Lima, Alexandre Morrot

**Affiliations:** ^1^Instituto de Microbiologia, Universidade Federal do Rio de Janeiro, Rio de Janeiro, Brazil; ^2^Laboratório de Glicobiologia, Instituto de Biofísica Carlos Chagas Filho, Universidade Federal do Rio de Janeiro, Rio de Janeiro, Brazil

**Keywords:** glycosylation, immunesurveillance, cancer immunology, immune evasion, tumor virulence

## Abstract

Glycans are part of the essential components of a cell. These compounds play a fundamental role in several physiopathological processes, including cell differentiation, adhesion, motility, signal transduction, host–pathogen interactions, tumor cell invasion, and metastasis development. Glycans are also able to exert control over the changes in tumor immunogenecity, interfering with tumor editing events and leading to immune-resistant cancer cells. The involvement of glycans in cancer progression is related to glycosylation alterations. Understanding such changes is, therefore, extremely useful to set the stage for their use as biomarkers, improving the diagnostics and therapeutic strategies. Herein, we discuss the basis of how modifications in glycosylation patterns may contribute to cancer genesis and progression as well as their importance in oncology field.

## Glycosylation as an Essential Protein Post-Translational Modification

**Figure 1:**
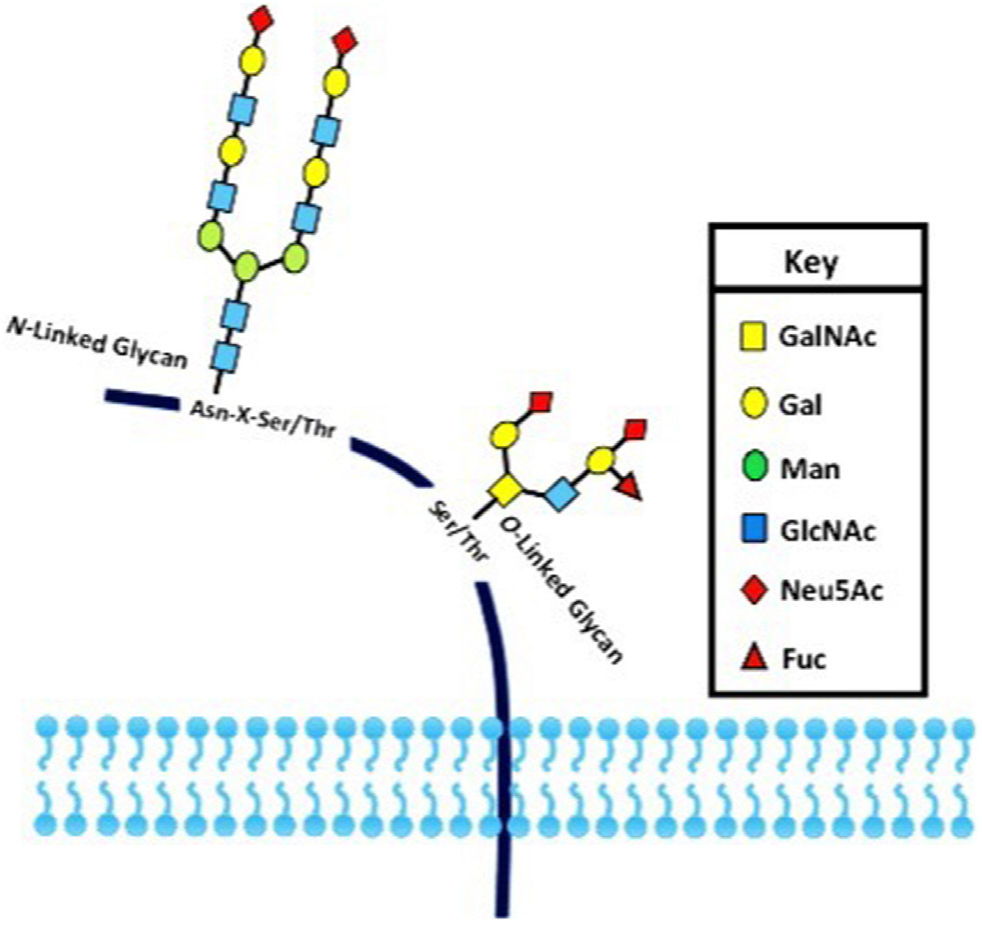
**The two major types of protein glycosylation**. The attachment of sugar moieties to proteins is a post-translational modification that provides greater proteomic diversity to the proteins. N-linked glycosylation occurs through the asparagine residues of the protein, while O-linked glycosylation occurs through serine or threonine.

Post-translational modifications (PTMs) exert an important role in controlling protein function in eukaryotes. PTMs comprise processes such as acetylation, carbonylation, methylation, hydroxylation, nitration, palmitoylation, phosphorylation, sulfation, ubiquitination, and glycosylation (1, 2). It is universally accepted that deregulation of such PTMs may lead to the development of a number of diseases. The glycosylation is the most common PTM and occurs in all domains of life. As a result, they impart an additional level of “information content” to underlying protein structures (3, 4). Two basic types of protein glycosylations are *N*- and *O*-glycosylations (Figure 1) with significant differences in terms of their biosynthesis and structures, as well as their location within the protein chain (5). In *N*-linked glycans, the nitrogen atom in the side chain of asparagine is attached to *N*-acetylglucosamine (GlcNAc). The sequence can be Asn–X–Ser or Asn–X–Thr, where X is any kind of amino acid except proline. In O-linked glycans, the oxygen atom in the side chain of serine or threonine is attached to *N*-acetylgalactosamine (GalNAc) (5). Furthermore, the glycopeptides Asn-GlcNAc or Ser/Thr-GalNAc may be extended by numerous and specific glycosyltransferase activities (6, 7) (Figure 1).

## Altered Tumor-Cell Glycosylation Promotes Cancer Virulence

During the past few years, we have seen a breakthrough in understanding the molecular and cellular mechanisms of immune cell activation and homeostasis. Several studies have shown that glycosylation orchestrates important features in several pathological processes, mainly in cancer development and/or progression (8–11). Extensive progresses in defining the cellular and molecular networks that regulate the immune responses against different sort of tumors have renewed our enthusiasm to search for potential cancer immunotherapies. However, the successful translation of novel mechanistic insights into effective tumor immunotherapy is hindered by a number of obstacles, including the ability of tumor cells to generate a tolerant microenvironment (12–15). The immune response is crucial not only to elicit protection against pathogens but also to maintain immune surveillance against the development of malignant cells. From this perspective, the development of cancer can be seen as a failure of immune surveillance (16–18). The notion that the immune system can recognize and extinguish developing transformed cells was originally exemplified by the Burnet and Thomas’ hypothesis concerning the mechanisms of cancer immune surveillance (19, 20). This hypothesis supports that the immunoediting of tumor antigens is responsible for sculpting the immunogenic phenotypes of transformed cells that eventually induce immunocompetent hosts. However, most tumor-associated carbohydrate antigens (TACAs) do not elicit strong humoral responses and, in fact, pieces of evidence have shown that the aberrant expression of glycan structures, as well as occurrence of truncated structures, precursors, or novel structures of glycan might prevent effective immune responses against tumor cells (21–23). Some TACAs with immunomodulatory or immunosuppressive properties are presented in Table 1.

**Table 1:** **Glycan types and their main role in the subversion of antitumor immune responses**.

Glycan type	Glycan structure	Enzyme	Immunobiological effect	Reference
*N*-linked glycan	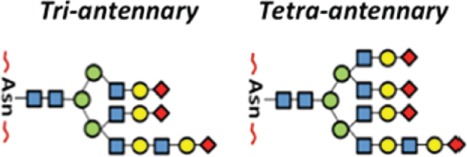	ã β1,6 GlcNAc-T *(MGATS)*	Helping the growth of cancer cells through inactivation of CD4^+^ T cells and macrophages	(24)
*O*-linked glycan	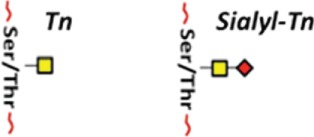	ä β1,4 Gal-T	Enhancing the production of anti-inflammatory cytokines; inducing a tolerogenic phenotype in innate and adaptive immune cells	(25, 26)
		ä COSMC		
*N*-linked glycan	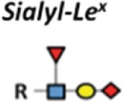	ã α 1,3 Fuc-T III	Potentiating cancer metastasis; leading to lung tumor formation or rejection by NK cells	(27–29)
*O*-linked glycan				
*N*-linked glycan	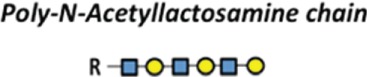	*N*-glycan ã β1,6 GlclMAc-T	Resistance against NK cell attack, promoting tumor metastasis	(30–33)
*O*-linked glycan		*O*-glycan ã Core 2GnT		
*N*-linked glycan	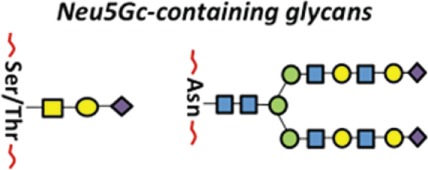	Neu5Gc is not synthesized in humans, it is incorporated into human tissues from dietary sources. Different sialyltransferases can use Neu5Gc as substrate	Neu5Gc-containing glycans are recognized as foreign antigens by the immune system and induce chronic inflammation	(34–37)
*O*-linked glycan				
Key	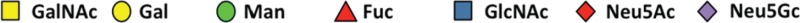			

## Tumor-Associated Glycan Determinants Subvert Key Immunological Defense Mechanisms

The first demonstration that tumor cells express altered glycans came from studies showing that monoclonal antibodies may recognize such abnormal structures (38). Many of these alterations are accompanied by expression of oncofetal antigens on tumor glycoproteins as well as glycosphingolipids (38, 39). Furthermore, it is well known that modifications in glycan structures may contribute to early stages of invasion (40–42), although it is not clear if such alterations may also play a role in the genesis of neoplastic cells (43, 44).

### Glycosphingolipids as Immunosuppressive Components in Tumor Cell Progression

Glycosphingolipids might also be involved in tumor cell progression by causing immune silencing. It has been also described that different types of cancer cells occasionally secrete gangliosides into the bloodstream (45, 46). In spite of their immunogenic properties, shaded membrane sialoglycolipids (gangliosides) may cause inhibition of co-stimulatory molecules synthesis, thus, promoting an impairment of dendritic cell (DC) maturation, leading to immune silencing by means of the inability of these cells to arm effective antitumor T cells responses (47, 48). A promising antitumor therapeutic approach using glycosphingolipids relies on therapeutic manipulation of these molecules to generate passive as well as active immunity (49–51).

### Role of Endogenous Lectins Blocking the Adaptive Immunity Against Tumor Cells

Alterations in the glycosylation profile in tumor compared to health cells are mainly attributed to the gene expression deregulation of glycosyltransferases, enzymes responsible to transfer sugars from donor to acceptor substrate molecules, leading to the synthesis of immature core glycans (52, 53). Specific glycans may bind to cell surface lectins such as galectins, C-type lectins, and siglecs [sialic acid (Sia)-binding immunoglobulin-type lectins], resulting in the regulation of cancer immune responses due to interference with the tumor immunoediting, characterized by changes in the immunogenicity of target antigens that could favor the dissemination of cancer cells (54–56). Galectins are β-galactoside-binding proteins that share homology in the amino acid sequence of their carbohydrate-recognition domain. Their role in immune responses against tumor cells has been studied over the past years (57–59).

In cancer, galectins secreted by tumor cells exhibit tolerogenic effects over effector T cells, promoting a cytokine imbalance that can either result in T cell anergy or favoring T regulatory (T reg) cell activity (60, 61). This immune modulation leading to tolerogenic responses against tumor cells can be associated with galectin-1 expression (62). The galectin-3, another member of galectin family, is known to induce apoptosis of antitumor CD8^+^ T cells (CTLs) in murine model of colorectal cancer (63). In addition, galectin-3 was demonstrated to increase the distance between the TCR and CD8 molecule in human CTLs infiltrating the tumors, a matter that turn them to be anergic (64). Besides their role in attenuating adaptive immune responses, galectins are also described to be able to impair the antitumor functions of natural killer (NK) cells (54).

The C-type lectins constitute by far the largest family of animal lectins found as part of membrane proteins and in soluble forms, comprising L-selectin, P-selectin, and E-selectin glycoproteins (65–67). These molecules act promoting the adhesion of leukocytes to the vascular endothelium, recognizing sialyl Lewis X (SLeX), sialyl Lewis A (SLeA), and Sia found in *O*-linked glycans (68). Such Lewis carbohydrate antigens can be also found on tumor cells as part of mucin glycoproteins and selectins that play a major role in migration processes by binding to endothelium during tissue infiltration along tumor metastasis (69). In murin models, mice displaying deficiency in L- and P-selectins presented a reduction of the metastisation (70). In addition, specific intercellular adhesion molecule-3 grabbing non-integrin 1 expressed on DCs (DC-SIGN), another transmembrane protein belonging to the C-type lectin family, is expressed by DCs and binds to aberrant *O*-glycosylation structures on cancer cells (71, 72). These abnormal glycoconjugates expressed by tumor cells are also able to interact with other class of receptors on DCs as well as macrophages, known as macrophage galactose-type C-type lectin, which are able to induce cellular cytotoxicity (73).

### Role of Sialic Acid Domains in Avoiding Cell-Mediated Immunity Against Tumor Cells

Immune cells continually screen the glycan structures that are expressed on cell surface glycoproteins from pathogen and host cells. The Sias are part of these multiple cell surface carbohydrates (74). The Sia motifs are differently expressed among the species, thus, allowing the immune system to distinguish *self* from *non-self*. In this sense, pathogens evolved to express Sia molecules mimicking the one present in host cells, therefore, subverting the host immunity (75, 76). The idea of Sia as self-associated molecular patterns (SAMPs) came from the fact that they may elicit inhibitory signals in order to prevent inadequate immune responses (77). Nonetheless, a growing body of evidence has been pointed out to an extensive contribution of sialoglycan motifs to tumor immune subversion (78, 79).

A multiplicity of ways whereby Sia molecules contribute to immune evasion mechanisms has been described. Complement system has evolved as a first line of defense against non-self or invading pathogens (80, 81). In neoplastic transformation, inhibition of complement activation allows the tumor cells to escape from immune responses (82). In fact, lung cancer cells and glioblastomas, for instance, are able to produce the inhibitory complement factor H, thus avoiding their elimination (83, 84). Tumor cells may evade the complement system activity through binding of Sia motifs present on their surface to polyanionic sites of the complement factor H component, thus activating a complement negative regulatory pathway (85).

Sialoglycans also play a role in tumor immunity mediated by NK cells, which are able to recognize transformed cells due to the lack of MCH class I molecules. However, tumor cells in turn may express inhibitory receptors impairing the cytotoxicity of NK cells (85). The presence of a dense layer of sialoglycans on tumor cell surfaces avoids the normal occurrence of immunological synapses between cancer and NK cells. Such reduced recognition is believed to be enhanced by hypersialylation of tumor ligands for the CD94/NKG2 family of transmembrane C-type lectin-like receptors (NKG2D) expressed by not only NK cells but also NK1.1^+^ T cells, γδ T cells, and activated CD8^+^αβ T cells and macrophages (86). The NKG2D receptors specifically recognize self-proteins from MIC and RAET1/ULBP families induced on the surface of stressed, malignant transformed, and infected cells (87). The hypersialylation of tumor ligands is thought to repulse their interaction with NKG2D receptors via highly negative charge (88).

The tumor-derived sialoglycans can also affect antitumor functions of NK cells via Sia/siglecs binding (89, 90). In fact, it has been shown that the immunomodulatory effects of tumor cells in part have influence of interactions between sialoglycans derived from transformed cells and the immune inhibitory siglec receptors (69). In fact, studies have demonstrated that overexpression of siglec ligands in tumor cells leads to impairment of the protective immune responses elicited by NK cells and neutrophils (91, 92). In addition, blockade of siglec-9 improved antitumor neutrophil responses *in vitro* (92). Siglec receptor triggering with sialylated mucins derived from tumor cells is able to induce inhibitory signals to immune cells, a process that is thought to be associated with tumor progress (41).

Tumor-derived sialoglycans can target different aspects of the immune system to promote evasion responses. It has been shown that tumor-derived Sia inhibits the traffic and subsequent exocytosis of lytic granules from CTLs to the immunological synapse, disabling a key mechanism whereby these lymphocytes eradicate tumor cells (93). Moreover, Sias have also been described to take part in the hypersialylation process of Fas receptor (CD95) on tumor cells, damping its binding to the Fas-ligand (CD95L) expressed by CTLs (94). The blockage of CD95/CD95L interaction impairs the downstream activation of caspases and consequently disarming the apoptosis machinery that would lead to tumor cell elimination (69). In this context, hypersialylation of Fas receptor by upregulation of sialyltransferase ST6Gal-I in tumor cells has also been described (95).

Besides its effects on cytotoxic T cells, tumor-derived sialoglycans are also able to dampen DC functions (96–100). Studies have shown that tumor-derived sialogangliosides inhibit the upregulation of co-stimulatory molecules (CD80/CD86) as well as IL-12 production by DCs, thus impairing T cell effector lymphocyte activation (101). This immunosuppressive effect is thought to be elicited by the interaction of highly sialylated tumor antigens with the siglec receptors expressed by DCs (102, 103). Moreover, the interaction of tumor-derived sialylated antigens with siglec receptors expressed by macrophages has also been described as a mechanism influencing tumor progression (104). In these studies, siglec-9 expressed by macrophages is shown to induce high levels of the immunosuppressive IL-10 cytokine together with reduction of the pro-inflammatory TNF-α cytokine associated with antitumoral responses (105).

## Concluding Remarks

Cell surface glycosylation patterns may suffer important changes during pathological conditions, especially in tumor invasion processes. Such alterations are the result of genetics as well as epigenetics changes, conferring to the tumor cells the ability of dissemination by escaping the immunesurveillance mechanisms (10, 106). This immune evasion phenomenon is being clarified, pointing out an important role for the shield created by altered sialylated glycans on the surface of cancer cells on the subversion of the immune system. This evolutionary conserved strategy can also be observed in some pathogens such as trypanosomatids, bacteria, and fungi (57). Understanding how abnormal glycosylation patterns effectively contribute to tumor-induced immune deviation would lead to early detection of potential tissue alterations and ultimately the development of therapeutic approaches against cancer.

## Author Contributions

AN, LF-L, CGF-L, and AM wrote the paper. All authors read and approved the final version of the manuscript.

## Conflict of Interest Statement

The authors declare that the research was conducted in the absence of any commercial or financial relationships that could be construed as a potential conflict of interest.
